# Maternal Zika virus exposure and neurodevelopmental outcomes: A longitudinal study of preschool children in the ZIKAlliance Colombian Cohort

**DOI:** 10.1371/journal.pone.0346805

**Published:** 2026-04-13

**Authors:** Víctor Herrera, María Consuelo Miranda, Anyela Lozano-Parra, Diana Niño, Luis Ángel Villar, Rosa Margarita Gélvez Ramírez, Thomas Jaenisch, Laura Pezzi, Claudia Acevedo, Jürg Niederbacher

**Affiliations:** 1 Grupo Epidemiología Clínica, Escuela de Medicina, Universidad Industrial de Santander, Bucaramanga, Colombia; 2 Centro de Atención y Diagnóstico de Enfermedades Infecciosas (CDI), Fundación INFOVIDA, Bucaramanga, Colombia; 3 Heidelberg Institute of Global Health, Heidelberg University Hospital, Heidelberg, Germany; 4 Center for Global Health, Colorado School of Public Health, Aurora, Colorado, United States of America; 5 National Reference Center for Arboviruses, Inserm-IRBA, Marseille, France; 6 Unité des Virus Émergents (UVE: Aix-Marseille Univ, Università di Corsica, IRD 190, Inserm 1207, IRBA), Marseille, France; 7 Grupo de Investigación de Pediatría (PAIDÓS), Departamento de Pediatría, Universidad Industrial de Santander, Bucaramanga, Colombia; Universidad Cooperativa de Colombia, COLOMBIA

## Abstract

**Background:**

Zika virus (ZIKV) infection has been inconsistently associated with neurodevelopmental delay (ND). We aimed to compare the incidence of ND between ZIKV-exposed and ZIKV-unexposed children within the ZIKAlliance (ZA) cohort, in Colombia, assessed 2 years after birth (2018–2021).

**Methods:**

We performed a neurodevelopmental evaluation on normocephalic children (aged 40–72 months) from the ZIKAlliance cohort. Children were classified as ZIKV-exposed (maternal positive RT-qPCR or virus neutralization test – VNT) or unexposed (maternal negative IgG ELISA or VNT in paired antenatal samples). A trained psychologist, blinded to exposure status, administered the Denver Developmental Screening Test II (DDST-II). Children were considered at ND risk if they presented ≥1 delay or ≥2 cautions in one or more areas, within their age range in the DDST-II scale. Inconclusive initial tests were re-evaluated. Adjusted odds ratios were estimated using logistic regression.

**Results:**

We analyzed conclusive DDST-II results from 153 children (mean age: 4.7 years; 53.8% male). Overall, 57.2% (n = 83) were classified as cases of ND. Children with ND were more likely to be male (61.4% versus 43.5%) and less likely to attend daycare or school (42.2% versus 11.3%) than children with normal development. After adjusting for child age, sex, household size, and education, the association between in utero ZIKV exposure and ND was not statistically significant (OR = 0.71; 95% CI: 0.32–1.59, p = 0.320). However, children attending daycare or school had a significantly lower risk of ND compared to those who stayed at home.

**Conclusions:**

Prenatal ZIKV exposure was not associated with ND in this cohort of normocephalic preschool children. Instead, attending a community daycare or school emerged as a significant protective factor against developmental delays.

## Introduction

The Zika virus (ZIKV), a member of the *Flavivirus* genus within the *Flaviviridae* family, is primarily transmitted by *Aedes m*osquitoes. While often asymptomatic, ZIKV infection can cause mild illness with symptoms like fever, headache, joint pain, rash, and conjunctivitis, mimicking other arboviral diseases like dengue and chikungunya. However, during pregnancy, ZIKV poses a significant threat, potentially leading to fetal microcephaly and other central nervous system (CNS) malformations. Additionally, ZIKV infection has been linked to autoimmune disorders like Guillain-Barré syndrome [1].

The alarming rise in microcephaly cases and potential associations with ZIKV led the World Health Organization (WHO) to declare a Public Health Emergency of International Concern (PHEIC) in 2016 [2]. This decision was prompted by the outbreak of severe microcephaly and other CNS malformations in newborns in northeast Brazil during 2014–2015, a condition termed Congenital Zika virus Syndrome (CZS) [3,4]. Microcephaly, alongside cortical malformations, ventriculomegaly, and cerebellar/brainstem hypoplasia, are hallmarks of CZS. These children often experience additional challenges like visual impairments, movement limitations (arthrogryposis), and neurodevelopmental delays [5–7].

During the ZIKV outbreak in 2015–2017, Brazil and Colombia were the most affected countries in Latin America reporting 369,013 and 108,730 cases of the infection, respectively [8]. Together, these countries accounted for over 3,200 cases of CZS, with Brazil reporting 92.2% of these cases [8]. Despite extensive research on CZS, there is a gap in knowledge regarding the long-term neurodevelopmental impact of prenatal exposure to ZIKV infection, particularly in children who appear clinically normal at birth. While some studies suggest a higher risk of neurodevelopmental delays in seemingly exposed children [9,10], others report no significant differences compared to unexposed controls [11–13]. These discrepancies may be related to the heterogeneity in follow-up duration, the neurodevelopmental assessment tools and the design or composition of the control group used.

Given the critical role of early childhood in brain development, even subtle prenatal lesions from infections like ZIKV can manifest as neurodevelopmental delays later in life. This study represents an extension of the ZIKAlliance (ZA) cohort, EU-funded consortium (Grant number 734548) aiming to evaluate the risk of congenital malformations (with the goal of further characterizing CZS) and other adverse pregnancy outcomes in women infected with ZIKV during pregnancy, stratified by gestational age at the time of infection [14]. Our goal for this initial extension of the ZikAlliance (ZA) cohort was to address a knowledge gap regarding the long-term neurodevelopmental effects of prenatal Zika virus (ZIKV) exposure. We investigated the incidence of neurodevelopmental impairments in preschool-aged children who were normocephalic at birth and whose mothers were pregnant during the Zika outbreak in Colombia. This was achieved by directly comparing the neurodevelopmental outcomes between children with and without in utero ZIKV exposure.

## Materials and methods

### Study design and participants

This is a cohort study nested within the ZA multi-center cohorts (Pregnant women [ZA-PW] and children [ZA-CH] cohorts) established during the Zika virus outbreak in several Latin American countries [14,15]. The ZA multi-center cohorts were designed to estimate the absolute and relative risks of congenital abnormalities and adverse outcomes associated with ZIKV infection during pregnancy [14]. In Colombia, the cohort recruited women in their first trimester of pregnancy who attended prenatal care between June 2017 and November 2019 at three primary healthcare centers located in the north of Bucaramanga (Socioeconomic strata 1). Pregnant women (PW) underwent monthly follow-up evaluations until delivery and one month postpartum. Then, the newborns were followed up at 1–3, 4–6, 12, and 24 months postpartum. The ZA multi-center cohorts collected demographic, socioeconomic, epidemiological, and clinical data, including mother health, substance exposure, and neonatal complications/findings at birth. Infants’ neurodevelopment status was routinely assessed through the Ages and Stages Questionnaire 3rd edition (ASQ-3) at 1–3, 4–6, 12, and 24 months [16]. A cranial ultrasound and a visual and auditory screening test were performed on the children aged 1–3 and 4–6 months [14]. The results of the follow-up during the first two years, along with the data from the entire ZIKAlliance cohort, will be published separately.

### Exposure

The exposure was assessed using the Euroimmun anti-ZIKV ELISA IgG assays (Euroimmun, Lübeck, Germany) on paired samples from all PW at enrollment and delivery, with sensitivity and specificity of 97.1% and 100%, respectively, following manufacturer’s instructions [17]. Samples from PW with suspected ZIKV symptoms were additionally tested through RT-qPCR (reference Centers for Disease Control and Prevention. Real Time RT-PCR for Detection of Zika Virus; Molecular Diagnostics and Research Laboratory, Division of Vector-Borne Diseases, CDC Dengue Branch: San Juan, Puerto Rico, 2015) during the acute phase of the illness. IgG ELISA and RT-qPCR tests were performed at the Universidad Industrial de Santander in Colombia, and those mothers with positive results on both assays were further confirmed by a viral neutralization test (VNT) conducted at Unité des Virus Emergents (UVE) (Marseille, France). Children born to mothers with a positive RT-qPCR test or at least one positive VNT result during pregnancy were considered ZIKV-exposed (dilution ≥ 1/40). In contrast, children born to mothers with negative anti-ZIKV ELISA IgG results in paired samples were considered as ZIKV-unexposed. Children whose mothers had positive anti-ZIKV ELISA IgG results but without VNT confirmation were classified as ZIKV-indeterminate.

### Eligibility and follow-up

Children with confirmed ZIKV exposure and at least one postpartum follow-up within the Colombian ZA cohort were eligible for additional neurodevelopmental evaluations. All participants in this group had undergone comprehensive clinical assessments at birth or during the first two years of life to rule out any structural abnormalities, especially microcephaly. These assessments included transfontanellar ultrasound and auditory and visual evoked potentials, all performed within the first four months of age. Given the significant time (two years) that had elapsed since the last contact with the parents or legal guardians of the participants in the ZA-CH cohort, we implemented a comprehensive re-contact protocol to maximize participation in the new follow-up neurodevelopmental assessment. This protocol included multiple phone calls, text messages, social media searches, and household visits. We could not include children residing outside Bucaramanga or those whose parents or legal guardians were unavailable to provide consent for the follow-up evaluation.

Eligible children and their parents or legal guardians were invited to an in-person visit to obtain informed consent and undergo comprehensive clinical and neurodevelopmental evaluations. These evaluations were conducted between November 24, 2022, and November 7, 2023, by a trained physician and psychologist, respectively. The clinical evaluation included an updated medical history, a thorough physical examination, anthropometric measurements, and a detailed neuromotor function, gait, and language assessment to identify any previously undetected abnormalities. Parents and evaluators were blinded to the ZIKV exposure status. In the first case, blindness to the exposure followed a recommendation by the Ministry of Health, according to which only the National Reference Laboratory or designated collaborators could inform Zike test results. Furthermore, it was not until the first quarter of 2024 that laboratory testing to determine the ZIKV exposure status of participants in the ZIKAlliance cohorts was completed.

### Outcomes

#### Denver developmental screening test, second edition (DDST-II).

We evaluated children using the Spanish version of the DDST-II, a screening test designed to identify children at risk for developmental disorders across personal-social, fine motor, gross motor, and language areas [18]. This version has been successfully evaluated regarding criterion validity, reliability, and internal consistency [19]. The test administration took an average of 20 minutes, with the number of activities or items assessed varying based on the child’s age and abilities, ranging in this study from 25 to 42.

The items to be evaluated for each child were determined by drawing an “age line” on the paper answer sheet indicating the test’s starting point. Then, the items for each area were administered at increasing difficulty levels. For interpreting the items, a “delay” was considered if a child failed or refused to perform an item completed by over 90% of children in their age group in the standardization sample. A “caution” was recorded if the child failed or refused to perform an item that was achieved by 75–90% of children of the same age. The DDST-II was interpreted as normal if there were no delays and at most one caution; as suspicious if there were one or more delays and/or two or more cautions; and as untestable if a child refused to perform one or more items successfully completed by over 90% of children of his/her age or two or more items completed by 75–90% of children of his/her age.

We repeated the DDST-II within 1–2 weeks if a test result was deemed untestable, after giving the caregiver a plan of home activities targeted at the areas where the child failed. These activities were designed by a neurodevelopment expert and provided to caregivers regardless of their children’s ZIKV exposure status. The study#39;s primary outcome was neurodevelopmental delay (ND), defined as a suspicious DDST-II test in its first (and unique) or second administration when required. Children with untestable results in both the first and second administrations of the test were excluded from the analysis. Secondary outcomes were defined as the DDST-II area-specific scores, which were computed by summing the number of items successfully completed based on the child#39;s age and the number of items up to the test’s starting point [20].

#### Ages and stages questionnaire, third edition (ASQ-3).

We alternatively also evaluated children’s development progress using the 60-month ASQ-3 questionnaire, whose suggested range of age for application is 57−66 months. This screening tool was administered to parents or caregivers in a controlled environment, by a trained evaluator to evaluate children’s performance in the communication, fine motor, gross motor, problem-solving, and socio-individual domains. Each item in each domain was assigned 10, 5, or 0 points if the parent or caregiver’s answer to the corresponding performance question was “yes”, “sometimes”, or “not yet”, respectively. The score for each domain was calculated as the sum of points over all the items (maximum: 60 points). Each domain of the ASQ-3 was classified as “below the expectation” if the corresponding score was less than the age-dependent thresholds [16].

### Ethical statement

The Ethics Committee of the Universidad Industrial de Santander approved the study protocol (Acta No.15, September 09, 2022), which had previously approved the conduct of the ZA-PW and ZA-CH cohort studies (Acta No. 05, 21 March 2017 and Acta No. 29, December 15, 2017, respectively). Children’s parents or legal guardians provide written informed consent to participate in compliance with Resolution 8430 of the Ministry of Health of Colombia. Also, the study staff instructed parents or legal guardians to consult their children’s healthcare provider if any abnormal finding was detected during the physical or neurodevelopmental evaluation.

### Data analysis

We described continuous variables by estimating the mean and standard deviation (SD) or the median and interquartile range [IQR] for those not normally distributed, according to the Shapiro-Wilk test. We calculated their absolute and relative frequencies (percentages) for discrete variables. We compared means and medians between groups using a student’s t-test and the sum of ranks test, respectively, and differences in proportions by using the chi-square test and, alternatively, the Fisher’s exact test whenever the expected counts in contingency tables were less than five. We estimated the odds ratio (OR) and 95% confidence interval (95%CI) of ND between ZIKV-exposed and ZIKV-unexposed children using logistic regression, adjusting for confounders. These variables were initially identified as maternal or child factors with p-values < 0.200 in the bivariate analysis. Using a forward-selection approach, they were retained in the regression model because they either significantly contributed to predicting the outcome (p-value < 0.05) or, when excluded, changed the magnitude of the exposure coefficient by more than 10%. Linearity in the logit for continuous predictors was assessed using the Box-Tidwell approach by including interaction terms between each predictor and its natural logarithm. We evaluated the model’s goodness of fit using the Hosmer-Lemeshow test and checked for multicollinearity by estimating the variance inflation factor (VIF). The same approach was implemented to determine the association between exposure to ZIKV and abnormal (“below the expected”) results for each domain of the ASQ-3 test. In addition, we assessed differences in scores across each DDST-II area between children exposed and unexposed to ZIKV using multiple linear regression, adjusting for relevant confounders as previously defined. Linearity between continuous covariates and the outcome was assessed using residual-versus-fitted-value plots, and, when heteroskedasticity was present (as determined by the Breusch-Pagan test), we estimated standard errors using a robust method. Finally, we checked for multicollinearity in each model by estimating the VIF. The analysis was conducted using Stata version 12.0 (Stata Corp.).

## Results

**Fig 1:**
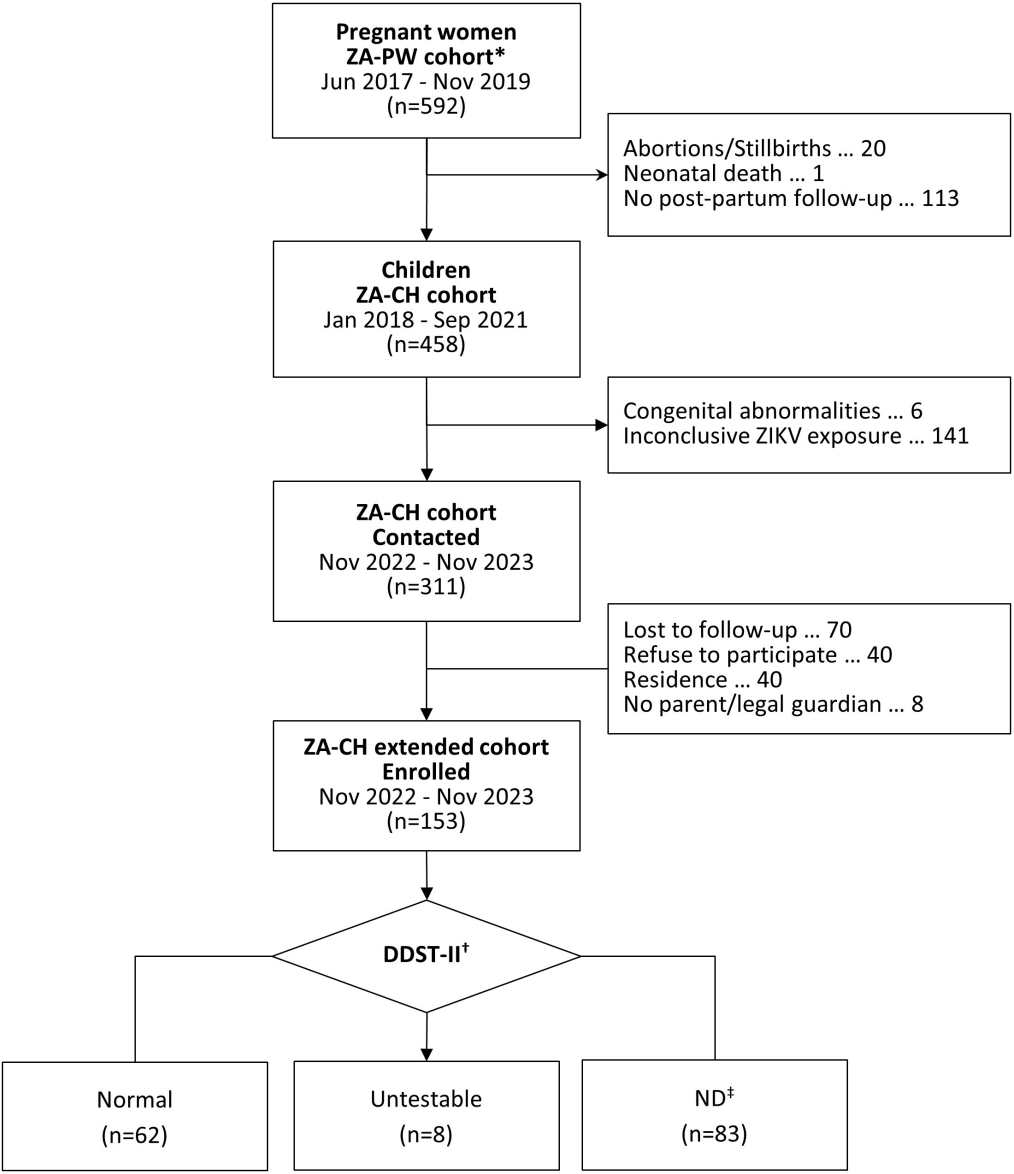
Flowchart of the pregnant women (PW) and children (CH) original and extended Zikalliance cohorts. *There were 590 women with 588 single and two multiple (twin) pregnancies. ^†^Denver Developmental Screening Test, second edition. ^‡^Neurodevelopmental delay (ND).

The ZA-PW cohort enrolled 590 women with 588 single and two multiple (twin) pregnancies (Fig 1). After the exclusion of 20 abortions and stillbirths, one neonatal death, and 113 newborns whose parents or legal guardians did not consent to further follow-up, the ZA-CH original cohort included 458 participants. We additionally excluded six children with a diagnosis of congenital abnormalities and 141 with an inconclusive ZIKV exposure before implementing the re-contact protocol for ZA-CH extended cohort study. This protocol allowed us to contact and enroll 153 children (49.2%) who were not different from those lost to follow-up, refused to participate, resided outside the study area, or whose parents or legal guardians could not consent, except by a slightly higher maternal body mass index and likelihood of requiring a resuscitation maneuver at birth (S1 and S2 Tables).

We evaluated 153 children between November 2022 and November 2023, 145 (94.8%) of which had a valid DDST-II assessment (mean age = 4.7 years, SD = 0.5 years; 53.8% male): 138 (95.2%) required only one while 7 (4.8%) required two assessments. There were 62 (42.8%) children with a normal DDST-II test and 83 (57.2%) who were classified as cases of ND. These groups were not statistically different in terms of maternal characteristics (Table 1); however, compared to normal children, those with ND were more likely males (61.4% versus 43.5%) and stay at home instead of attending a community daycare or school during the follow-up evaluation (42.2% versus 11.3%; Table 2). Importantly, all children from both groups had a normal head circumference and neurological examination at birth. Additionally, 7 (4.8%) children underwent a second DDST-II (re-test): 2 (3.2%) and 5 (6.0%) of those classified as having and without delayed neurodevelopment, respectively.

**Table 1:** Pregnant women’s baseline and follow-up characteristics by neurodevelopmental status.

Characteristic	Neurodevelopmental status		p-value
	Normal (n = 62)	Delayed (n = 83)	
Enrollment			
Maternal age (years)	23.3 [8.4]	22.7 [7.8]	0.554
Weight (kg)	61.5 [16.5]	61.0 [18.5]	0.569
Height (m)	1.57 (0.05)	1.57 (0.05)	0.898
BMI (kg/m^2^)	24.5 [9.1]	24.4 [6.6]	0.536
Persons per room	1.5 [1.0]	1.5 [0.8]	0.142
Education			0.374
Elementary	36 (58.1)	47 (56.6)	
Highschool	25 (40.3)	30 (36.1)	
Undergraduate or higher	1 (1.6)	6 (7.2)	
Household income*			0.572
≤1	54 (88.5)	71 (87.7)	
2	7 (11.5)	8 (9.9)	
≥3	0 (0.0)	2 (2.4)	
Alcohol consumption			0.756
Never	45 (72.6)	63 (75.9)	
Former	14 (22.6)	18 (21.7)	
Current	3 (4.8)	2 (2.4)	
Smoking			0.751
Never	47 (75.8)	65 (78.3)	
Former	13 (21.0)	17 (20.5)	
Current	2 (3.2)	1 (1.2)	
Recreational drugs			0.537
Never	57 (95.0)	69 (94.5)	
Former	1 (1.7)	0 (0.0)	
Current	2 (3.3)	4 (5.5)	
Comorbidities			
Gestational diabetes	6 (9.7)	10 (12.2)	0.790
Preeclampsia	4 (6.5)	4 (4.8)	0.724
Follow-up			
Persons per room	2.0 [0.9]	1.7 [0.8]	0.050
Socioeconomic status^†^			0.374
Low	36 (58.1)	47 (56.6)	
Middle	25 (40.3)	30 (36.1)	
High	1 (1.6)	6 (7.2)	
Maternal education			0.526
Elementary	10 (16.1)	14 (16.9)	
Highschool	35 (56.5)	53 (63.9)	
Undergraduate or higher	17 (27.4)	16 (19.2)	

Figures in each cell correspond to mean (standard deviation), median [interquartile range], or absolute (relative) frequencies. *Number of household’s monthly income in minimum legal wage(s). ^†^Classified according to the National Administrative Department of Statistics (DANE) as low (socioeconomic strata I-II), middle (socioeconomic strata III-IV), and high (socioeconomic strata V-VI).

**Table 2:** Children#39;s characteristics at birth and extended follow-up by neurodevelopmental status.

Characteristic	Neurodevelopment status		p-value
	Normal (n = 62)	Delayed (n = 83)	
Birth			
Gestational age (weeks)	39.0 [2.6]	39.0 [3.3]	0.397
Male	27 (43.5)	51 (61.4)	0.043
Weight (gr)	3210.9 (369.9)	3132.9 (484.9)	0.295
Crown-heel (cm)	49.9 (1.6)	49.9 (2.1)	0.991
Head circumference (cm)	33.9 (1.3)	33.8 (1.5)	0.669
APGAR score (minute)			
1^st^	8.0 [0.0]	8.0 [0.0]	0.611
5^th^	10.0 [1.0]	9.0 [1.0]	0.965
10^th^	10.0 [0.0]	10.0 [0.0]	0.237
Complications/findings			
Resuscitation	3 (4.9)	3 (3.6)	0.698
Jaundice	2 (3.4)	4 (5.1)	1.000
Abnormal physical exam			
Head	0 (0.0)	0 (0.0)	–
Face	0 (0.0)	0 (0.0)	–
Eyes	0 (0.0)	0 (0.0)	–
Cardiovascular	1 (1.7)	0 (0.0)	0.428
Respiratory	0 (0.0)	2 (2.5)	0.507
Gastrointestinal	0 (0.0)	0 (0.0)	–
Genital	0 (0.0)	0 (0.0)	–
Limbs	0 (0.0)	0 (0.0)	–
Neurological exam			
Neck tonic reflex	60 (100.0)	78 (100.0)	–
Moro reflex	59 (100.0)	79 (100.0)	–
Sucking reflex	59 (100.0)	79 (100.0)	–
Grasp reflex	60 (100.0)	79 (100.0)	–
Hypotonia	0 (0.0)	0 (0.0)	–
Paralysis	0 (0.0)	0 (0.0)	–
Limb paralysis	0 (0.0)	0 (0.0)	–
Stiffness	0 (0.0)	0 (0.0)	–
Follow-up			
Age (years)	4.8 (0.5)	4.7 (0.5)	0.139
DDST-II re-test	2 (3.2)	5 (6.0)	0.699
Education/care*			<0.001
Home	7 (11.3)	35 (42.2)	
Communitarian daycare	26 (41.9)	32 (38.6)	
School	29 (46.8)	16 (19.3)	

Figures in each cell correspond to mean (standard deviation), median [interquartile range], or absolute (relative) frequencies. *Children might stay home, attend communitarian daycares, or attend the formal school system.

There was no difference in the probability of prenatal exposure to ZIKV between children with a normal DDST-II test and those with ND: 53.2% versus 42.2%, respectively (OR=0.64; 95%CI: 0.33–1.25; Table 3). This finding was confirmed by the multivariate analysis after controlling for children’s age, sex, household overcrowding (persons per room), and exposure to educational environments at follow-up (adjusted OR=0.71; 95%CI: 0.32–1.59). The latest was the only factor associated with ND, showing an inverse dose-response relationship: children who attended a community daycare and a school were 71.0% (OR=0.29; 95%CI: 0.11–0.77) and 90.0% (OR=0.10; 95%CI: 0.03–0.33) less likely to be classified as cases of ND than those staying at home, respectively. Notably, we did not observe a difference in the mean scores of any domain of the Ages and Stages Questionnaire evaluated before the current follow-up visit between children who stayed at home and those who attended a community daycare or school (S3 Table).

**Table 3:** Crude and adjusted odds ratios (OR) of neurodevelopmental delay by Zika exposure status.

Predictor	Model	
	Unadjusted	Adjusted
Zika-exposed vs. unexposed	0.64 (0.33, 1.25)	0.71 (0.32, 1.59)
Age (years)	–	1.42 (0.62, 3.28)
Male	–	2.12 (0.99, 4.52)
Persons per room	–	0.92 (0.53, 1.62)
Education/care*		
Home	–	1.00
Communitarian daycare	–	0.29 (0.11, 0.77)
School	–	0.10 (0.03, 0.33)

*Children might stay home, attend communitarian daycares, or attend the formal school system. No significant deviations from linearity were observed (p > 0.05) for any continuous predictor. Multivariate (adjusted) model’s Hosmer-Lemeshow p-test = 0.320. VIF < 2.0 for all covariates, with an average VIF of 1.36.

ZIKV-exposed children did not differ from unexposed children in terms of DDST-II’s area-specific mean scores either: 24.7 versus 24.8 points in the personal-social area, 28.6 versus 28.5 points in the fine motor area, 31.3 versus 31.0 points in the gross motor area, and 37.8 versus 37.8 points in the language area. These results were not changed by adjusting for confounders (S4 Table). Regarding the ASQ-3 test, there were no differences in the proportion of results “below the expectation” for any domain between children exposed and unexposed to ZIKV after controlling for confounders (Table 4).

**Table 4:** ASQ-3 domain-specific proportion of results “below the expected” by Zika exposure status.

Domain	Exposed	Non-exposed	OR* (95%CI)
Communication	2 (2.9)	0 (0.0)	–
Fine motor	11 (16.2)	8 (10.4)	1.16 (0.37, 3.66)
Gross motor	4 (5.9)	7 (9.1)	0.62 (0.13, 2.98)
Problem-solving	2 (2.9)	6 (7.8)	0.10 (0.01, 1.06)
Socio-individual	1 (1.5)	2 (2.6)	0.16 (0.01, 2.88)

* Adjusted for children’s age, sex, household overcrowding, and exposure to educational environments during the follow-up. No significant deviations from linearity were observed (p > 0.05) for any continuous predictor. P-values > 0.05 for the Hosmer-Lemeshow tests of all models. VIF < 2.0 for all covariates, with an average VIF of 1.36.

## Discussion

In this study, which extended the follow-up of the children’s ZIKAlliance cohort, we did not find a significant association between prenatal ZIKV exposure and neurodevelopmental delay (ND) as assessed by the Denver II (DDST-II) and Ages and Stages Questionnaire, Third Edition (ASQ-3). On the other hand, we did observe that children attending a community daycare or school were at lower risk of neurodevelopmental delay as compared to those who remained at home.

Our findings align with previous research suggesting that normocephalic children exposed to ZIKV may not have a significantly increased risk of neurodevelopmental delay [11–13,21–23]. Although results comparing ZIKV-exposed and unexposed preschool/school-age infants are limited and have shown mixed results [9,24], our findings contribute to the expanding body of evidence in this area. However, making direct comparisons across studies remains challenging due to differences in the criteria for defining exposure, selecting unexposed groups, and the neurodevelopmental testing tools used. In contrast, the conflicting results reported by Mulkey et al. [9,24], our findings are consistent in demonstrating no significant difference between ZIKV-exposed and unexposed children, regardless of the neurodevelopmental assessment employed (DDST-II or ASQ-3), and represent a result of a more rigorous methodological approach. Specifically, whereas Mulkey et al., relied on the date of conception in relation to the national epidemic curve to rule out exposure and included non-concurrent unexposed cohorts, our study determined exposure status through laboratory testing. Moreover, although losses to follow-up were substantial in both studies, we have provided evidence refuting the notion that such attrition introduced selection bias. Finally, in our study, neither the parents of children nor the outcome evaluators were aware of the exposure status, a highly desirable design feature lacking in Mulkey’s cohorts, thereby minimizing the likelihood of differential parental behavioral and attitudinal changes based on the knowledge of their children’s exposure, as well as information bias.

The DDST-II represents a versatile instrument for large-scale developmental assessments. It has been validated against the gold-standard Bayley Scales of Infant and Toddler Development (third edition, BSDI-III), demonstrating strong predictive capacity for outcomes in middle childhood, thereby rendering it particularly valuable in situations where comprehensive testing is impractical [20]. Although the DDST-II has previously been employed to evaluate neurodevelopment in children with microcephaly exposed to ZIKV during pregnancy [25–28], to the best of our knowledge, this study is the first to implement this tool to test the hypothesis of an association between prenatal exposure to ZIKV and neurodevelopmental delay among preschool and school-aged normocephalic children. Notably, our findings, based on the DDST-II, are consistent with those reported in other cohorts that implemented the Bayley-III, although among children aged 24–42 months [11–13] which altogether strongly argue against prenatal ZIKV exposure as a risk factor for neurodevelopmental delay in normocephalic children. Furthermore, as previously discussed, we concurrently evaluated neurodevelopment using the DDST-II and the ASQ-3, yielding consistent results. Regarding the latest test, our results were similar to those reported by Grant et al. [21], although in their cohort study, neurodevelopment was evaluated in children aged 2 years.

Our findings also suggest a link between children#39;s exposure to educational environments and neurodevelopmental delays. Children attending a community daycare or a school had a significantly lower risk of neurodevelopmental delay than those who stayed at home. This gradient may be attributed to the emphasis on play-based learning, art, literature, and environmental exploration in the Colombian Institute of Family Welfare#39;s community daycare programs [29]. Additionally, previous studies indicate that high-quality childcare and early childhood education settings have a positive impact on neurodevelopment in low- and middle-income countries (LMICs) [30,31]. Alternatively, our findings may be due to selection bias, in which case, children at higher risk of neurodevelopmental delay were less likely to have access to a daycare or a school; however, this explanation seems unlikely considering the absence of difference in the neurodevelopmental trajectories, based on the ASQ-3, of children attending a daycare or a school compared to those who stayed at home in our cohort.

Our study provides valuable insights into the potential long-term effects of prenatal ZIKV exposure on child neurodevelopment and has some strengths worth mentioning. Firstly, the extended follow-up period allowed an unprecedented longitudinal evaluation of the neurodevelopment among normocephalic children in Colombia. Secondly, the inclusion of a well-matched control group within the ZIKAlliance cohort allowed for a direct comparison between exposed and unexposed children, minimizing selection bias. Thirdly, the use of the DDST-III, administered by a trained psychologist with both the evaluator and parents blinded to ZIKV exposure status, minimized the risk of information bias in assessing the association between exposure and neurodevelopment. On the other hand, our study also has some limitations. First, we were unable to definitively determine the exact time of ZIKV exposure, which may have led to an underestimate of the association mainly due to the incorrect assignment of prenatal exposure to children born to mothers with pre-conceptional exposure. Secondly, although the DDST-II has been previously validated, it is primarily a screening test for detecting potential neurodevelopmental delays rather than a diagnostic tool, with reported sensitivity and specificity of 89% and 92%, respectively [19]. In the absence of sensitivity and specificity estimates by strata of ZIKV exposure, and under the assumption of non-differential misclassification of the outcome, we expect an underestimation of the magnitude of the association between ZIKV exposure and neurodevelopmental delay. Thirdly, the extended cohort had a high rate of losses to follow-up; despite this, children lost to follow-up were comparable to those who were followed in terms of not only baseline sociodemographic and clinical characteristics but also, more importantly, regarding their neurodevelopmental trajectories, as longitudinally evaluated using the ASQ-3. These similarities between exposed and unexposed children suggest a non-informative mechanism driving losses to follow-up, therefore, a low risk of selection bias.

## Conclusions

Neurodevelopmental outcomes among normocephalic children exposed to ZIKV in utero continue to be an area of active investigation, particularly as these children reach school age. In our study, based on a well-characterized cohort assembled during the Zika epidemic in Colombia, we found no evidence of an association between prenatal exposure to ZIKV and neurodevelopmental delay, as independently assessed using two different scales. This finding aligns with the results from the majority of cohorts investigated in this field.

## Supporting information

S1 Table Baseline* characteristics of mothers of children who were lost or followed for neurodevelopmental evaluation. (DOCX)

S2 Table Baseline* characteristics of children who were lost or followed for neurodevelopmental evaluation. (DOCX)

S3 Table Mean score of the ASQ domains* by exposure to educational environments. (DOCX)

S4 Table Mean score of the DDST-II domains* by exposure to ZIKV. (DOCX)
