# Supplementary material for: Maternal Zika virus exposure and neurodevelopmental outcomes: A longitudinal study of preschool children in the ZIKAlliance Colombian Cohort
Source: PLoS One. 2026 Apr 13;21(4):e0346805. doi: 10.1371/journal.pone.0346805 (PMC13075668; doi:10.1371/journal.pone.0346805)
Supplement: S1 Table — (DOCX) [file pone.0346805.s001.docx]

**S1 Table. Baseline* characteristics of mothers of children who were lost or followed for neurodevelopmental evaluation.**

| **Characteristic** | **Follow-up** | | **p-value** |
| --- | --- | --- | --- |
|  | **Lost**  **(n=158)** | **Followed**  **(n=153)** |  |
| Gestational age (weeks) | 14.3 (5.9) | 14.7 (6.3) | 0.609 |
| Age (years) | 24.5 (5.8) | 24.2 (5.6) | 0.649 |
| Weight (kg) | 59.3 (12.7) | 62.8 (13.4) | 0.023 |
| Height (m) | 1.58 (0.05) | 1.57 (0.05) | 0.171 |
| BMI (kg/m^2^) | 23.9 (4.9) | 25.5 (5.4) | 0.006 |
| Persons per room | 1.7 (1.0) | 1.6 (0.7) | 0.206 |
| Education attainment |  |  | 0.369 |
| Elementary | 25 (15.8) | 27 (17.7) |  |
| Highschool | 122(77.2) | 109 (71.2) |  |
| Undergraduate or higher | 11 (7.0) | 17 (11.1) |  |
| Household income^†^ |  |  | 0.436 |
| ≤1 | 130 (83.3) | 132 (88.0) |  |
| 2 | 24 (15.4) | 16 (10.7) |  |
| ≥3 | 2 (1.3) | 2 (1.3) |  |
| Alcohol consumption |  |  | 0.456 |
| Never | 126 (79.8) | 113 (73.9) |  |
| Former | 29 (18.4) | 35 (22.9) |  |
| Current | 3 (1.8) | 5 (3.2) |  |
| Smoking |  |  | 0.860 |
| Never | 118 (74.7) | 116 (75.8) |  |
| Former | 35 (22.2) | 34 (22.2) |  |
| Current | 5 (3.1) | 3 (2.0) |  |
| Recreational drugs |  |  | 0.901 |
| Never | 147 (98.6) | 134 (95.0) |  |
| Former | 1 (0.7) | 1 (0.7) |  |
| Current | 1 (0.7) | 6 (4.3) |  |
| Previous pregnancies (n) | 1.3 (1.3) | 1.3 (1.4) | 0.915 |
| Obstetric complications^‡^ |  |  |  |
| Gestational diabetes | 11 (7.0) | 18 (11.8) | 0.173 |
| Preeclampsia | 5 (3.2) | 9 (5.9) | 0.284 |
| ZIKV exposure | 61 (38.6) | 71 (47.3) | 0.135 |
| *Evaluated at enrollment of the ZA-PW cohort. ^†^Number of household’s monthly income in minimum legal wage(s). ^‡^Obstetric Complications were retrospectively evaluated at birth. | | | |
