# Supplementary material for: Maternal Zika virus exposure and neurodevelopmental outcomes: A longitudinal study of preschool children in the ZIKAlliance Colombian Cohort
Source: PLoS One. 2026 Apr 13;21(4):e0346805. doi: 10.1371/journal.pone.0346805 (PMC13075668; doi:10.1371/journal.pone.0346805)
Supplement: S2 Table — (DOCX) [file pone.0346805.s002.docx]

**S2 Table.** **Baseline* characteristics of children who were lost or followed for neurodevelopmental evaluation.**

| **Characteristic** | **Follow-up** | | **p-value** |
| --- | --- | --- | --- |
|  | **Lost**  **(n=158)** | **Followed**  **(n=153)** |  |
| Gestational age (weeks) | 39.2 (2.1) | 39.0 (2.6) | 0.527 |
| Male | 77 (48.7) | 87 (54.2) | 0.365 |
| Birth weight (gr) | 3181.9 (428.3) | 3165.4 (442.3) | 0.738 |
| Crown-heel (cm) | 49.5 (2.3) | 50.0 (1.9) | 0.076 |
| Head circumference (cm) | 34.0 (1.5) | 33.8 (1.4) | 0.450 |
| APGAR score (minute) |  |  |  |
| 1^st^ | 8.1 (0.4) | 8.0 (0.7) | 0.237 |
| 5^th^ | 9.6 (0.6) | 9.5 (0.6) | 0.161 |
| 10^th^ | 10.0 (0.1) | 9.9 (0.2) | 0.132 |
| Complications/findings |  |  |  |
| Resuscitation | 0 (0.0) | 6 (3.9) | 0.013 |
| Jaundice | 5 (3.2) | 7 (4.9) | 0.561 |
| Abnormal physical exam |  |  |  |
| Head | 1 (0.6) | 0 (0.0) | 1.000 |
| Face | 1 (0.6) | 0 (0.0) | 1.000 |
| Eyes | 1 (0.6) | 0 (0.0) | 1.000 |
| Cardiovascular | 0 (0.0) | 1 (0.7) | 0.480 |
| Respiratory | 2 (1.3) | 2 (1.4) | 1.000 |
| Gastrointestinal | 0 (0.0) | 0 (0.0) | - |
| Genital | 0 (0.0) | 0 (0.0) | - |
| Limbs | 0 (0.0) | 0 (0.0) | - |
| Neurological exam |  |  |  |
| Neck tonic reflex | 155 (100.0) | 144 (100.0) | - |
| Moro reflex | 156 (100.0) | 144 (100.0) | - |
| Sucking reflex | 156 (100.0) | 144 (100.0) | - |
| Grasp reflex | 156 (100.0) | 145 (100.0) | - |
| Hypotonia | 0 (0.0) | 0 (0.0) | - |
| Paralysis | 0 (0.0) | 0 (0.0) | - |
| Limb paralysis | 0 (0.0) | 0 (0.0) | - |
| Stiffness | 0 (0.0) | 0 (0.0) | - |
| Ages and Stages Questionnaire† |  |  |  |
| Communication | 53.0 (5.6) | 53.3 (5.5) | 0.576 |
| Fine motor | 51.7 (5.8) | 52.0 (4.8) | 0.627 |
| Gross motor | 53.4 (6.7) | 52.7 (7.3) | 0.383 |
| Problem-solving | 51.3 (6.0) | 52.5 (5.1) | 0.060 |
| Socio-individual | 52.1 (5.3) | 51.0 (5.9) | 0.092 |
| *Evaluated at enrollment of the ZA-CH cohort (birth). † Mean of scores reported before the current follow-up. | | | |
