# Supplementary material for: Maternal Zika virus exposure and neurodevelopmental outcomes: A longitudinal study of preschool children in the ZIKAlliance Colombian Cohort
Source: PLoS One. 2026 Apr 13;21(4):e0346805. doi: 10.1371/journal.pone.0346805 (PMC13075668; doi:10.1371/journal.pone.0346805)
Supplement: S3 Table — (DOCX) [file pone.0346805.s003.docx]

**S3 Table. Mean score of the ASQ domains* by exposure to educational environments.**

| **Domain** | **Home** | **Community daycare** | **School** | **p-value** |
| --- | --- | --- | --- | --- |
| Communication | 52.4 (6.0) | 53.7 (5.1) | 53.7 (5.4) | 0.430 |
| Fine motor | 51.3 (5.2) | 52.8 (4.6) | 51.6 (4.7) | 0.239 |
| Gross motor | 53.1 (8.3) | 53.2 (6.5) | 51.6 (7.4) | 0.501 |
| Problem-solving | 51.5 (5.6) | 52.9 (4.7) | 53.1 (5.0) | 0.268 |
| Socio-individual | 50.3 (6.7) | 51.9 (5.1) | 50.3 (6.0) | 0.226 |
| * Mean of scores reported before the current follow-up visit. | | | | |
