# Supplementary material for: Maternal Zika virus exposure and neurodevelopmental outcomes: A longitudinal study of preschool children in the ZIKAlliance Colombian Cohort
Source: PLoS One. 2026 Apr 13;21(4):e0346805. doi: 10.1371/journal.pone.0346805 (PMC13075668; doi:10.1371/journal.pone.0346805)
Supplement: S4 Table — (DOCX) [file pone.0346805.s004.docx]

**S4 Table. Mean score of the DDST-II domains* by exposure to ZIKV.**

| **Domain** | **ZIKV-unexposed**  **(n=77)** | **ZIKV-exposed**  **(n=68)** | **Crude mean difference**  **(95%CI)** | **Adjusted* mean difference**  **(95%CI)** |
| --- | --- | --- | --- | --- |
| Personal-social | 24.8 (0.7) | 24.7 (0.7) | -0.1 (0.3, -0.2) | - |
| Fine motor | 28.5 (0.8) | 28.6 (0.7) | 0.1 (-0.1, 0.4) | 0.1 (-0.2, 0.4) |
| Gross motor | 31.0 (1.3) | 31.3 (1.0) | 0.3 (-0.1, 0.7) | 0.0 (-0.3, 0.3) |
| Language | 37.8 (1.3) | 37.8 (1.8) | 0.0 (-0.5, 0.5) | -0.1 (-0.7, 0.4) |
| * Model for the fine motor domain adjusted for child’s age and sex (global F-test: p=0.011; adjusted-R^2^=0.07; mean VIF=1.11); model for the gross motor domain adjusted by child’s age (global F-test: p=0.002; adjusted-R^2^=0.07; mean VIF=1.15); and model for the language domain adjusted by child’s age and care (global F-test: p=0.142; adjusted-R^2^=0.03; VIF=1.11). No relevant confounders were identified for the personal-social domain. Note: No significant deviations from linearity were observed for child’s age in any model. Moreover, due to heteroskedasticity standard errors were estimated using the robust method for all models. | | | | |
